# Supplementary material for: Regulation of substrate utilization and adiposity by Agrp neurons
Source: Nat Commun. 2019 Jan 18;10:311. doi: 10.1038/s41467-018-08239-x (PMC6338802; doi:10.1038/s41467-018-08239-x)
Supplement: Supplementary file 1 — Supplementary Information [file 41467_2018_8239_MOESM1_ESM.pdf]

Supplementary Material for “Regulation of substrate utilization and adiposity by Agrp neurons”

Cavalcanti-de-Albuquerque et al.

Supplementary Notes 1-3
Supplementary Figure 1-6
Supplementary References

Supplementary Note 1
We characterized the effects of glucose ingestion on metabolism. We provided different concentrations of glucose to mice via gavage delivery, thereby ruling out potential cephalic phase effects (**Supplementary Fig. S2a**). Gavage infusion of glucose led to dose-dependent increases in RER (**Supplementary Fig. S2b**; $r^2 = 0.99$, $P = 0.003$), decreases in fat utilization (**Supplementary Fig. S2c**; $r^2 = 0.99$, $P = 0.004$), and increases in carbohydrate utilization (**Supplementary Fig. S2d**; $r^2 = 0.99$, $P = 0.003$). These metabolic shifts were mainly due to increases in VCO_2 (**Supplementary Fig. S2f**; $r^2 = 0.98$, $P = 0.006$) and not VO_2 (**Supplementary Fig. S2e**; $r^2 = 0.84$, $P = 0.07$). Glucose intake also positively correlated with energy expenditure (**Supplementary Fig. S2g**; $r^2 = 0.92$, $P = 0.03$), likely due to the thermic effects of carbohydrate digestion. Activity levels were unchanged (**Supplementary Fig. S2d**; $r^2 = 0.58$, $P = 0.23$). These experiments demonstrate that small amounts of glucose ingestion alone are sufficient to shift metabolism even in the absence of carbohydrate sensing at the level of the mouth.

Supplementary Note 2
We micro-injected the arcuate nucleus of adult *Agrp*^{Cre} mice with an adeno-associated virus (AAV) carrying a *Cre*-dependent construct to express hM3Dq (AAV-DIO-hM3D(Gq)-mCherry; see Material and Methods and **Supplementary Fig. S3a**). We first confirmed the increased feeding response upon peripheral injection of clozapine-N-oxide in *Agrp*$^{\mathrm{hM3Dq}}$ mice (CNO, i.p.; **Supplementary Fig. S3b**). In indirect calorimetry chambers, activation of Agrp neurons in *Agrp*$^{\mathrm{hM3Dq}}$ mice led to metabolic shifts towards carbohydrate utilization related to fat utilization (**Supplementary Fig. S3d-j**), similarly to what we found in *Agrp*$^{\mathrm{Trpv1}}$ mice, but with a delayed latency as expected by the use of this tool [1,2] compared to Trpv1-mediated neuron activation [3,4,5].

Supplementary Note 3
Three weeks after the injection of an AAV carrying a conditional construct to express hM3Dq in *Agrp*^{Cre} mice, we confirmed mice responded to CNO injection by measuring their feeding response (**Supplementary Fig. 5a)**. After a period of washout, we switched mice to a high-fat diet for four days controlling their caloric ingestion (**Supplementary Fig. 5b**). Mice received daily injections of CNO (at ZT 11.0, i.p.). Similar to *Agrp*$^{\mathrm{Trpv1}}$ mice (**Fig. 6**), activation of Agrp neurons in *Agrp*$^{\mathrm{hM3Dq}}$ mice during controlled hyperphagia led to increased body weight gain (**Supplementary Fig. 5c**), metabolic efficiency (**Supplementary Fig. 5d**) and gain in fat mass (**Supplementary Fig. 5e**).

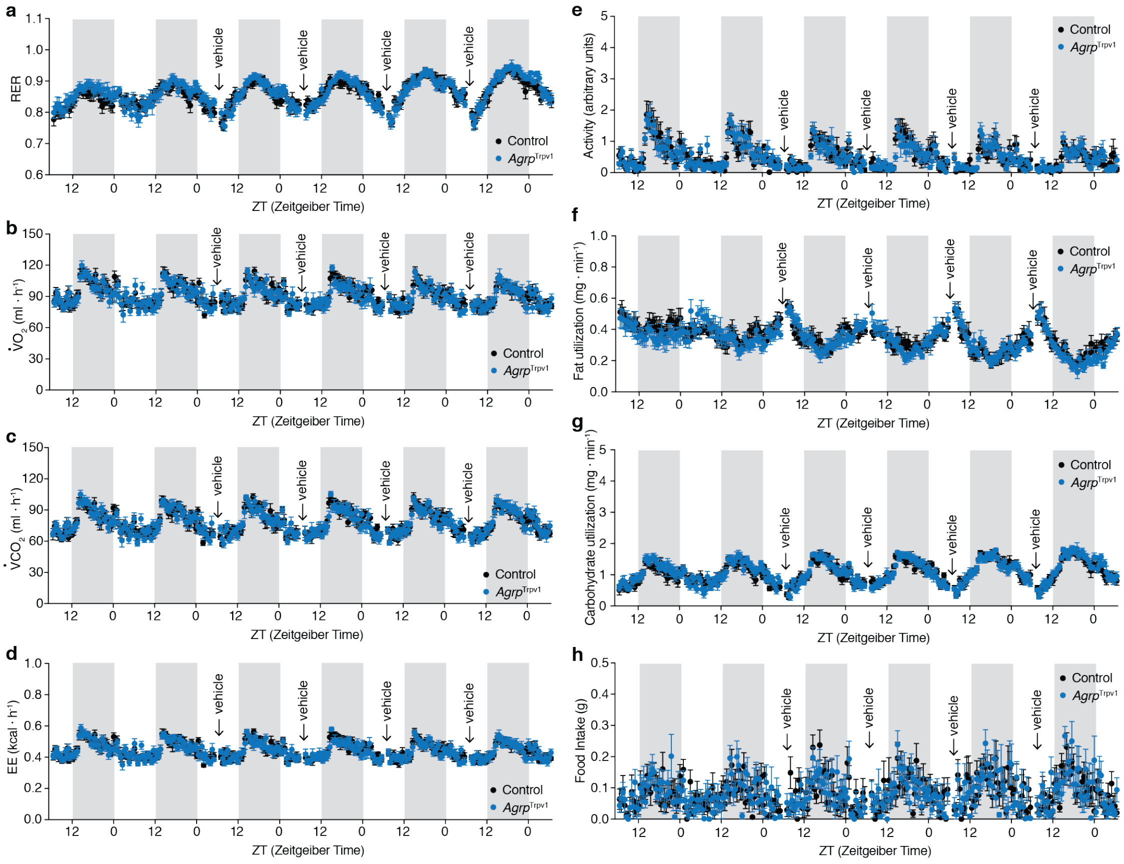

Supplementary Figure 1: Baseline calorimetry data in control and *Agrp*$^{\mathrm{Trpv1}}$ mice. Control (black; n = 8) and *Agrp*$^{\mathrm{Trpv1}}$ mice (blue; n = 8) were acclimated to indirect calorimetry chambers before injection with capsaicin. The panels represent the last two days of acclimation followed by 4 days of acclimation to vehicle injection (i.p.). Injections were performed during the light cycle at the same time as capsaicin injection (see Fig. 1). (**a**) RER. (**b**) VO2. (**c**) VCO2. (**d**) Energy expenditure. (**e**) Ambulatory activity. (**f**) Calculated fat utilization. (**g**) Calculated carbohydrate utilization. (**h**) Food intake. Grey shadows indicate dark cycle. Arrows indicate time of injection. Symbols indicate mean ± SEM.

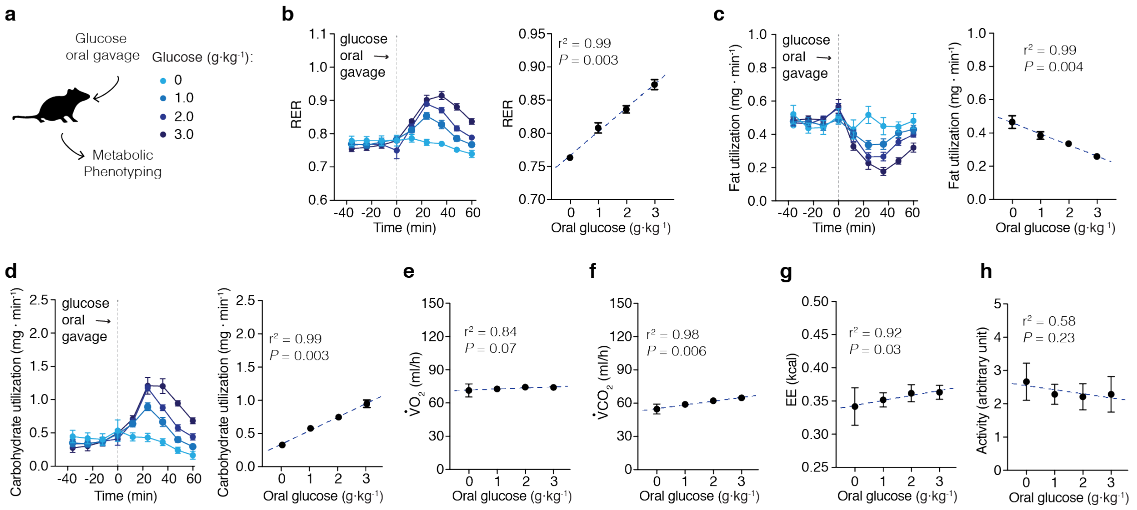

Supplementary Figure 2: Acute effects of glucose on metabolism.
(**a**) Mice received a bolus of saline or glucose solution (1, 2, or 3 g kg^{-1} body weight dissolved in saline) via gavage. (**b**) RER. (**c**) Calculated fat utilization. (**d**) Calculated carbohydrate utilization. (**e**) VO_2. (**f**) VCO_2. (**g**) Energy expenditure. (**h**) Activity levels. Saline (n = 7); glucose 1 g/kg (n = 12); glucose 2 g kg^{-1} (n = 16); and glucose 3 g kg^{-1} (n = 8). Symbols represent mean ± SEM. Grey dashed line indicates time of oral gavage. In the linear correlation panels, symbols indicate mean of all mice in the given group ± SEM; dashed red line represents the linear regression model; r^2 and P values are plotted in each panel.

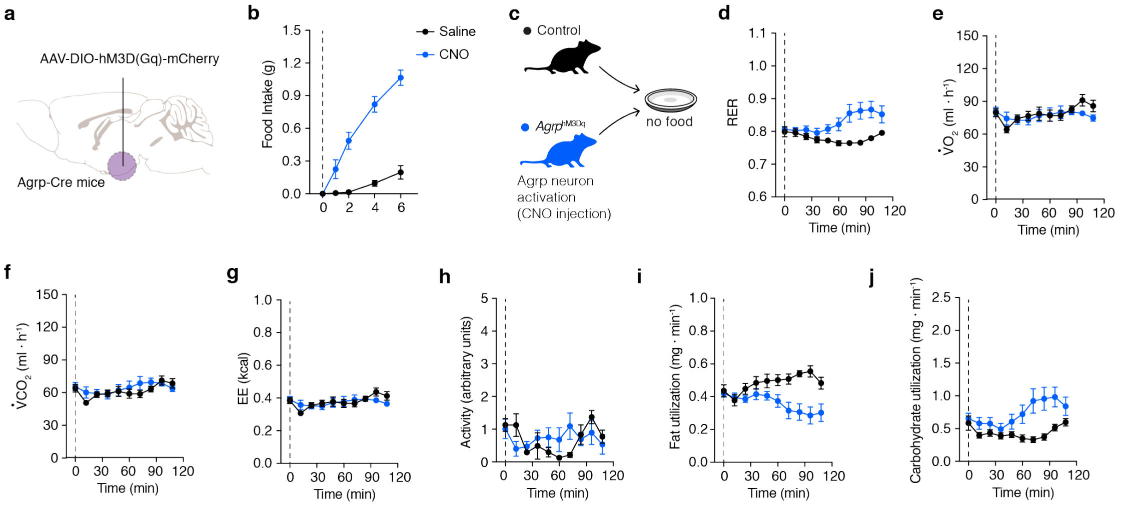

Supplementary Figure 3: Activation of Agrp neurons induces a shift in whole-body substrate utilization.

(**a**) An AAV-DIO-hM3D(Gq)-mCherry was injected in the arcuate nucleus of adult male *Agrp*^{Cre} mice. Animals were randomly injected with saline (n = 5) or CNO (1 mg kg^{-1}, i.p.; n = 5). (**b**) Food intake (interaction: $F_{4, 40}$ = 45.91, P < 0.0001; time: $F_{4, 40}$ = 94.51, P < 0.0001; and group: $F_{1, 10}$ = 60.36, P < 0.0001). (**c**) Control (*Agrp*^{hM3Dq} mice injected with saline) and *Agrp*^{hM3Dq} mice injected with CNO were tested in calorimetry chambers in the absence of food. (**d**) RER (interaction: $F_{9, 90}$ = 4.97, P < 0.0001; time: $F_{9, 90}$ = 3.07, P = 0.002; and group: $F_{1, 10}$ = 9.90, P = 0.01). (**e**) VO_2 (interaction: $F_{9, 90}$ = 2.32, P = 0.02; time: $F_{9, 90}$ = 4.44, P < 0.001; and group: $F_{1, 10}$ = 0.12, P = 0.73). (**f**) VCO_2 (interaction: $F_{9, 90}$ = 1.73, P = 0.09; time: $F_{9, 90}$ = 5.41, P < 0.0001; and group: $F_{1, 10}$ = 0.36, P = 0.56). (**g**) Energy expenditure (interaction: $F_{9, 90}$ = 2.17, P = 0.03; time: $F_{9, 90}$ = 4.72, P < 0.0001; and group: $F_{1, 10}$ = 0.02, P = 0.87). (**h**) Ambulatory activity (interaction: $F_{9, 90}$ = 1.90, P = 0.06; time: $F_{9, 90}$ = 1.96, P = 0.05; and group: $F_{1, 10}$ = 0.15, P = 0.70). (**i**) Calculated fat utilization (interaction: $F_{9, 90}$ = 6.53, P < 0.0001; time: $F_{9, 90}$ = 1.37, P = 0.21; and group: $F_{1, 10}$ = 9.97, P = 0.01). (**j**) Calculated carbohydrate utilization (interaction: $F_{9, 90}$ = 3.96, P = 0.0003; time: $F_{9, 90}$ = 4.60, P < 0.0001; and group: $F_{1, 10}$ = 7.30, P = 0.02). Statistical analysis was performed using two-way ANOVA with time as a repeated-measure followed by Holm-Sidak's multiple comparisons test (MCT). MCTs are not displayed in the figures. Dashed lines indicate time of saline or CNO injection. Symbols indicate mean ± SEM.

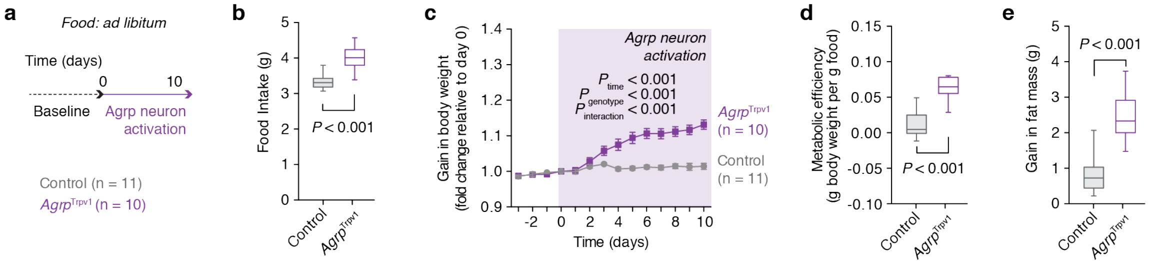

Supplementary Figure 4:
(**a**) Protocol used in the *ad libitum* studies; after baseline, control and *Agrp*$^{\text{Trpv1}}$ mice were injected with capsaicin (10 mg kg^{-1}; i.p.) for 10 days at 5:00 PM; food was provided ad libitum. (**b**) Average daily food intake over the 10-days period of capsaicin injection. (**c**) Gain in body weight relative to the first day of capsaicin injection. (**d**) Metabolic efficiency, calculated as the delta body weight minus total food intake during the 10-days period of capsaicin injection. (**e**) Gain in fat mass, calculated by the differences in fat mass after the period of neuron activation, using MRI to measure body composition. Two-way ANOVA with time as a repeated measure was used in (**c**). Unpaired *t* test was used in (**b**), (**d**) and (**e**). Number of mice is displayed in the figure. Symbols indicate mean ± SEM. Boxes indicate median ± 25/75 quartiles ± min/max values. *P* values are provided in the figures.

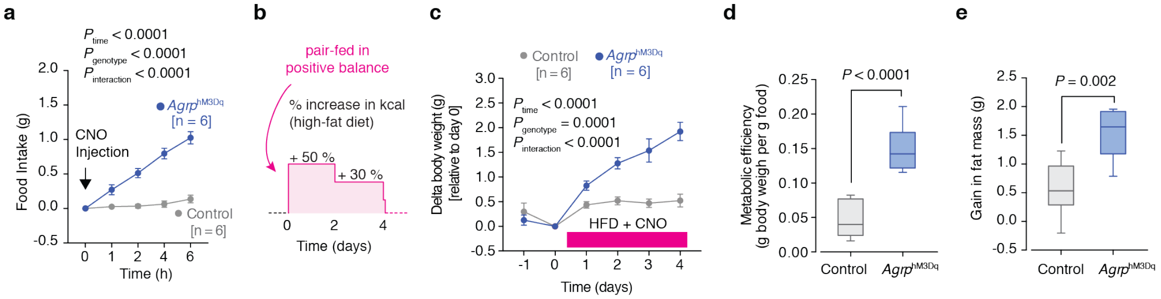

Supplementary Figure 5: Activation of Agrp neurons with hM3Dq recapitulates the effects of Agrp^{Trpv1} mice.
(**a**) Food intake after injection of CNO (0.3 mg kg^{-1}, i.p.) in control and *Agrp*^{hM3Dq} mice. (**b**) Mice were pair fed in positive energy balance for four days. (**c**) Delta changes in body weight relative to the day of diet switch. (**d**) Metabolic efficiency and (**e**) gain in fat mass during the period in which mice were fed high-fat diet. Two-way ANOVA with time as a repeated measure was used in (**a**) and (**c**). Unpaired *t* test was used in (**d**) and (**e**). Number of animals is provided in the panels. Symbols represent mean ± SEM. Boxes indicate median ± 25/75 quartiles ± min/max values.

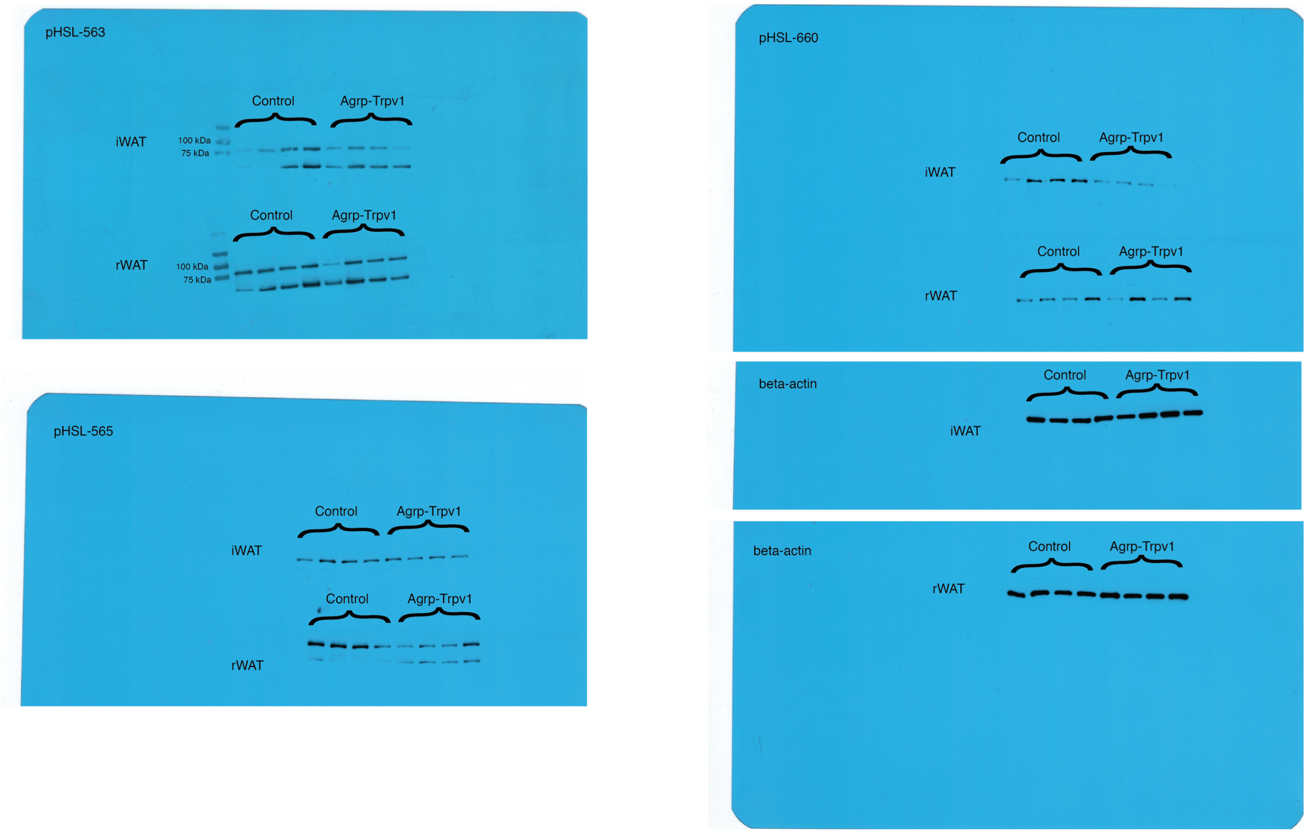

Supplementary Figure 6: Western blots.
Full size (uncut) western blot films.

Supplementary References

1. Krashes MJ*, et al.* Rapid, reversible activation of AgRP neurons drives feeding behavior in mice. *The Journal of clinical investigation* **121**, 1424-1428 (2011).

2. Krashes MJ, Shah BP, Koda S, Lowell BB. Rapid versus delayed stimulation of feeding by the endogenously released AgRP neuron mediators GABA, NPY, and AgRP. *Cell metabolism* **18**, 588-595 (2013).

3. Arenkiel BR, Klein ME, Davison IG, Katz LC, Ehlers MD. Genetic control of neuronal activity in mice conditionally expressing TRPV1. *Nature methods* **5**, 299-302 (2008).

4. Guler AD*, et al.* Transient activation of specific neurons in mice by selective expression of the capsaicin receptor. *Nature communications* **3**, 746 (2012).

5. Dietrich MO, Zimmer MR, Bober J, Horvath TL. Hypothalamic Agrp Neurons Drive Stereotypic Behaviors beyond Feeding. *Cell* **160**, 1222-1232 (2015).
