# Supplementary material for: Regulation of substrate utilization and adiposity by Agrp neurons
Source: Nat Commun. 2019 Jan 18;10:311. doi: 10.1038/s41467-018-08239-x (PMC6338802; doi:10.1038/s41467-018-08239-x)
Supplement: Supplementary file 3 — Reporting Summary [file 41467_2018_8239_MOESM3_ESM.pdf]

natureresearch

Corresponding author(s): Marcelo O. Dietrich

Reporting Summary

Nature Research wishes to improve the reproducibility of the work that we publish. This form provides structure for consistency and transparency in reporting. For further information on Nature Research policies, see Authors & Referees and the Editorial Policy Checklist.

Statistical parameters

When statistical analyses are reported, confirm that the following items are present in the relevant location (e.g. figure legend, table legend, main text, or Methods section).

n/a	Confirmed	
☐	☒	The exact sample size (*n*) for each experimental group/condition, given as a discrete number and unit of measurement
☐	☒	An indication of whether measurements were taken from distinct samples or whether the same sample was measured repeatedly
☐	☒	The statistical test(s) used AND whether they are one- or two-sided<br>*Only common tests should be described solely by name; describe more complex techniques in the Methods section.*
☐	☒	A description of all covariates tested
☐	☒	A description of any assumptions or corrections, such as tests of normality and adjustment for multiple comparisons
☐	☒	A full description of the statistics including central tendency (e.g. means) or other basic estimates (e.g. regression coefficient) AND variation (e.g. standard deviation) or associated estimates of uncertainty (e.g. confidence intervals)
☐	☒	For null hypothesis testing, the test statistic (e.g. *F*, *t*, *r*) with confidence intervals, effect sizes, degrees of freedom and *P* value noted<br>*Give P values as exact values whenever suitable.*
☒	☐	For Bayesian analysis, information on the choice of priors and Markov chain Monte Carlo settings
☒	☐	For hierarchical and complex designs, identification of the appropriate level for tests and full reporting of outcomes
☒	☐	Estimates of effect sizes (e.g. Cohen's *d*, Pearson's *r*), indicating how they were calculated
☐	☒	Clearly defined error bars<br>*State explicitly what error bars represent (e.g. SD, SE, CI)*

Our web collection on statistics for biologists may be useful.

Software and code

Policy information about availability of computer code

Data collection	*Provide a description of all commercial, open source and custom code used to collect the data in this study, specifying the version used OR state that no software was used.*
Data analysis	*Provide a description of all commercial, open source and custom code used to analyse the data in this study, specifying the version used OR state that no software was used.*

For manuscripts utilizing custom algorithms or software that are central to the research but not yet described in published literature, software must be made available to editors/reviewers upon request. We strongly encourage code deposition in a community repository (e.g. GitHub). See the Nature Research guidelines for submitting code & software for further information.

Data

Policy information about availability of data

All manuscripts must include a data availability statement. This statement should provide the following information, where applicable:

- Accession codes, unique identifiers, or web links for publicly available datasets
- A list of figures that have associated raw data
- A description of any restrictions on data availability

The data that support the findings of this study are available from the corresponding author upon reasonable request.

Field-specific reporting

Please select the best fit for your research. If you are not sure, read the appropriate sections before making your selection.

☒ Life sciences ☐ Behavioural & social sciences ☐ Ecological, evolutionary & environmental sciences

For a reference copy of the document with all sections, see nature.com/authors/policies/ReportingSummary-flat.pdf

Life sciences study design

All studies must disclose on these points even when the disclosure is negative.

Sample size	Samples sizes were not calculated but were estimated based on our preliminary results (Dietrich et al., Cell 2015) using similar animal models.
Data exclusions	No data were excluded.
Replication	All findings reported were replicated at least twice. For figure 7, we run a first study with n = 2 mice only. We made some minor changes in intra-gastric infusion rates and then replicated the study that is now reported.
Randomization	Subjects were randomly assigned for different treatment groups.
Blinding	For biochemical analysis, investigators were blinded for the experimental groups. For physiological analysis, all data were analyzed in batches, and subjected were assigned to groups post hoc.

Reporting for specific materials, systems and methods

Materials & experimental systems

n/a	Involved in the study	
☒	☐	Unique biological materials
☐	☒	Antibodies
☒	☐	Eukaryotic cell lines
☒	☐	Palaeontology
☐	☒	Animals and other organisms
☒	☐	Human research participants

Methods

n/a	Involved in the study	
☒	☐	ChIP-seq
☒	☐	Flow cytometry
☒	☐	MRI-based neuroimaging

Antibodies

Antibodies used	Rabbit-anti-AgRP (Pheonix Pharmaceuticals, cat. H-003-57); Donkey-anti-Rabbit Alexa Fluor 594 (Invitrogen, A21207)
Validation	Antibodies were validated by binding to brain regions that are known to express Agrp and staining was decreased with ablation of neurons producing the peptide.

Animals and other organisms

Policy information about studies involving animals; ARRIVE guidelines recommended for reporting animal research

Laboratory animals	Mice used in the experiments were 3 to 8 months old from both genders. The following mouse lines were used in this study: Agrptm1(cre)Lowl/J (Stock number 012899, Jax); Gt(ROSA)26Sortm1(Trpv1,ECFP)Mde/J (Stock number 008513, Jax); Trpv1tm1Jul/J (Stock number 003770, Jax); AgrpDTR (donated by Richard Palmiter).
Wild animals	The study did not involve wild animals.
Field-collected samples	The study did not involved collecting tissues in the field.
